# Photometric stereo for three-dimensional leaf venation extraction

**DOI:** 10.1016/j.compind.2018.02.006

**Published:** 2018-06

**Authors:** Wenhao Zhang, Mark F. Hansen, Melvyn Smith, Lyndon Smith, Bruce Grieve

**Affiliations:** a Centre for Machine Vision, Bristol Robotics Laboratory, University of the West of England, T Block, Frenchay Campus, Coldharbour Lane, Bristol, BS16 1QY, UK; b School of Electrical & Electronic Engineering, University of Manchester, Oxford Road, Manchester, M13 9PL, UK

**Keywords:** Leaf venation, Leaf disease, 3D imaging, Shape index, Photometric stereo, Ridge detection

## Abstract

Leaf venation extraction studies have been strongly discouraged by considerable challenges posed by venation architectures that are complex, diverse and subtle. Additionally, unpredictable local leaf curvatures, undesirable ambient illuminations, and abnormal conditions of leaves may coexist with other complications. While leaf venation extraction has high potential for assisting with plant phenotyping, speciation and modelling, its investigations to date have been confined to colour image acquisition and processing which are commonly confounded by the aforementioned biotic and abiotic variations. To bridge the gaps in this area, we have designed a 3D imaging system for leaf venation extraction, which can overcome dark or bright ambient illumination and can allow for 3D data reconstruction in high resolution. We further propose a novel leaf venation extraction algorithm that can obtain illumination-independent surface normal features by performing Photometric Stereo reconstruction as well as local shape measures by fusing the decoupled shape index and curvedness features. In addition, this algorithm can determine venation polarity – whether veins are raised above or recessed into a leaf. Tests on both sides of different leaf species with varied venation architectures show that the proposed method is accurate in extracting the primary, secondary and even tertiary veins. It also proves to be robust against leaf diseases which can cause dramatic changes in colour. The effectiveness of this algorithm in determining venation polarity is verified by it correctly recognising raised or recessed veins in nine different experiments.

## Highlights

• An accurate and robust leaf venation extraction method is proposed.
• The proposed 3D imaging system can recover illumination-independent and high-resolution surface normal features.
• The proposed venation extraction algorithm employs local shape measures by fusing shape index and curvedness features.
• The algorithm can determine venation polarity – whether veins are raised above or recessed into a leaf.
• The proposed method can overcome undesirable variations commonly found in real-world environments.

## 1 Introduction

Leaf venation is the patterned veins in the blade of a leaf. Leaf veins are considered to be related to resource delivery rates and photosynthetic capacity, but their high potential is undervalued and studies are limited [1]. Nelson in 1997 pointed out that the underlying mechanisms for leaf vascularisation were poorly understood and most studies were descriptive [2]. A few years later, computer vision studies centred on leaf venation extraction grew and developed, offering a transformative opportunity for quantitatively analysing leaf venation architectures [3]. Although this branch of computer vision studies is still at its youth, a few studies have shown that leaf venation extraction can greatly benefit plant speciation [4], phenotyping [5], as well as measuring physiological properties of plants (e.g. water transport and flow velocity in leaves [6]). It can even be of evolutionary significance by being able to reveal gene induced traits [[7], [8]].

However, in this emerging field, there has been limited work due to the considerable challenges, e.g. subtle vein structures that commonly have diameters less than one millimetre, complex vascularisation of veins, and other variations caused by biotic or abiotic stress and/or ambient lighting. Leaf venation architectures are very distinctive. Common venation types include *palmate venation* where primary veins radiate from the petiole, *pinnate venation* where secondary veins branch from a primary vein, *transverse venation* (or cross-venulate) where tertiary veins connect secondary veins, and *reticulated venation* where veins are net-like. Although leaves are extremely diverse in venation architecture, their venation systems commonly share two primary traits. Without explaining biological causes, these two traits can be summarised as:

1) Colour. Leaf veins and mesophyll have a certain level of difference in colour due to their varied reflectance properties. This can be characterised in images as edges and corners.
2) Topography. Leaf veins can appear to be ridge-like or rut-like structures relative to a leaf blade that is mostly locally flat.

While leaf veins can be a source of edges and corners in colour or greyscale leaf images, more edges may be caused by numerous factors including ambient illumination, local leaf curvatures, colour patterns of a leaf, leaf diseases, etc. In the rest of the paper, we refer to the former (leaf vein edges) as *true edges* and the latter as *false edges*. We also define the polarity of leaf venation according to whether they form ridges or ruts on a leaf blade − *positive* for ridge-like veins and *negative* for rut-like veins. It should be noted that, as each leaf has two sides (upper and lower epidermis), our definition of venation polarity is specific to either the upper side or the lower side of a leaf. For example, leaf venation can be negative on the upper side of a leaf, but positive on the lower side.

Regarding leaf venation architectures, differences in individuals and across species are so significant that they cannot be well characterised by image colour or intensity based features alone. Apart from inherent variations that pose great challenges to leaf venation extraction, environmental factors further magnify this by causing complications such as leaf dehydration, powdery mildew, leaf curl, leaf mottling, etc. Investigations of these different traits [9] in laboratory environments often enforce sufficient and homogeneous lighting to avoid presence of severe shadows and specularities. However, in real-world environments, shadows and specularities are commonly inevitable while undesirable illumination can also cause images to be underexposed or overexposed. Therefore, we propose a 3D leaf imaging system and a leaf venation extraction algorithm and we make the following contributions:

1) The proposed 3D leaf imaging system is capable of recovering illumination-independent and high-resolution surface normal (3D) features of leaves.
2) The proposed leaf venation extraction algorithm is fully compatible with the hardware system. It employs 3D features to realise wide applicability, high accuracy and robustness. It can also detect leaf vein polarity.
3) The proposed method can overcome undesirable variations commonly found in real-world environments such as illumination changes and abnormalities induced by leaf diseases.

## 2 Related work

In the last two decades, researchers have been attempting to resolve the leaf venation extraction problem by utilising different types of features in colour or greyscale images. We review in this section a number of representative methods and give an indication of the current research state as well as challenges being faced with and gaps to be bridged.

In 2003, [10] introduced a two-stage approach where intensity histogram information is initially used to filter out most background pixels. Gradient features representing edges which are further described by local contrast are combined with five statistical features based on intensity values. These features extracted from image regions are then used to train a neural network to achieve automatic classification of vein and non-vein pixels. The results showed that this method only achieved slightly better performance than using a Sobel filter [11]. Similar to this method, many others researchers also intuitively based their methods on edge features. For example, [12] proposed a leaf vein extraction method based on the active contour model. The active contour model [13], i.e. snakes, is widely used for image segmentation and edge detection, but it requires prior knowledge of desired contour shapes, i.e. characterised leaf vein structures in this case. Consequently, their method enforces definition of leaf vein geometries by comparing pixel colour and measuring pixel distance in the HSI colour space [14]. Due to these assumed characteristics of leaf venation, this method can only deal with a specific type of leaf venation architecture, and a high noise (non-vein pixels) level is still present in their demonstration of results. [15] investigated vein morphologies in greyscale images transformed in the HSV colour space. Morphological erosion and dilation, along with top-hat [16] and bottom-hat transformations, are employed by this method to obtain leaf venation. Disconnected vein segments are then jointed and isolated pixels removed. As their experiments were conducted on scanned leaf images (by a HP Scanjet 4070 photosmart scanner) where local leaf curvatures were flattened and illumination was near to ideal, this method would very likely suffer in dealing with leaf data captured in dynamic ambient environments.

A few other methods distinguish themselves by employing supervised or unsupervised learning methods to extract and process edge features differently. For example, [17] proposed to combine Sobel edges with an artificial neural network for leaf venation extraction. This method assumes that vein pixels are relatively darker than neighbouring pixels and extracts those around Sobel edges by comparing their first and second order derivatives. [18] presented a venation extraction method based on Independent Component Analysis to learn latent independent causes of leaf features by considering them as a set of linear basis functions. Results show that this method can detect primary and secondary veins of pinnate venations while tertiary veins will likely become noise.

As opposed to characterising leaf veins as edges, [19] considered them as ridges. In their work, the Hessian matrix for each pixel is calculated, which essentially consists of second-order derivatives of intensity values. This differs from many edge detection based methods by considering leaf veins as ridges instead of edges. By comparing the two Eigen values of a Hessian matrix, the local shape around each pixel can be quantified.

Recently, research works started to show how leaf venation could benefit plant speciation and suggested that this would further demand higher robustness against colour changes induced by factors such as diseases and nutritional deficiency. [20] proved the effectiveness of leaf venation combined with other features for plant recognition by achieving a recognition accuracy of 97.1% on a dataset with 1907 leaf images of 32 species. As they only employed basic morphological operations (i.e. erosion followed by dilation), only the primary vein and its direction could be determined. Its inherent limitations also mean that the venation extraction method cannot distinguish between true and false edges. [21] found that venation extraction on apple-tree leaves can, apart from benefiting plant recognition and plant growth analysis, assist with detection and localisation of discoloured leaf regions with different sizes and intensities, e.g. brown spots in the presence of hydrogen peroxide and plant peroxidases. The technique used for venation extraction is based on stepwise vein tracking at local image regions, which minimises a cost function designed for the pinnate venation architecture. As a result, it only tracks primary and secondary veins that are subject to a specific morphology and that do not change angle rapidly. Although this increases its robustness against image noise and leaf discolouration, this technique cannot be generalised for venation extraction of a variety of plant species and venation architectures. [22] pointed out that nutrition deficiency in plants will most likely lead to changes in interveinal areas and along the edges. Therefore, they proposed a method based on Canny edge detection. Thresholds were found heuristically to deal with edges of certain strength. This method was thus sensitive to pixel intensity variations, e.g. ambient lighting that can change strength of edges at a local or global level.

[23] showed that leaf vein image features could be used independently to achieve plant speciation with experiments on three legume species, namely soybean, red beans and white beans. This method applies a hit-or-miss transform on greyscale images, which extracts pixels that match a neighbourhood with a specific foreground and background pattern. Different classifiers, including the Support Vector Machine, Penalised Discriminant Analysis and Random Forest then utilise these features to recognise leaf species. It was claimed that this method could outperform manual experts’ recognition. More recently, they conducted another study [24] to increase the species recognition accuracy and to reveal the frequently used vein patterns. To achieve this, bag-of-words are employed to characterise leaf vein features represented by Scale-Invariant Feature Transform. Another extension of this work [25] employs a convolutional neural network for classification and improves recognition accuracy to 96.9%.

As can be seen from the review of the state-of-the-art leaf venation extraction methods, research in this area is still limited due to the following reasons:

1) All existing methods, to the best of our knowledge, are based on 2D features derived from colour or greyscale images.
2) The majority of methods (all except for one in those we reviewed) perform venation extraction on the lower side of a leaf blade where venation structures are prominent. However, acquisition of leaf images from a lower-side view is impractical in real-world environments.
3) Leaf data employed in the literature have all been captured under desirable lighting conditions.
4) Many methods are subject to predefined models that are not representative of all venations, e.g. they are only applicable to a specific type of venation architecture or they make assumptions that vein pixels are brighter or darker than their neighbouring pixels.

## 3 Photometric stereo for 3D leaf imaging

From the preceding sections, it can be concluded that the majority of leaf venation extraction methods in the literature only investigate colour traits by means of edge, corner and contour detection in individual colour or greyscale images. However, to the best of our knowledge, vein topography, i.e. the 3D surface, is left unexplored. Arguably, this is due to the vascular structure being highly inconspicuous, whose topography therefore needs to be characterised by high-resolution 3D data that can recover high-frequency 3D texture information. Acquisition of such data normally involves complex and expensive 3D imaging systems whereas inexpensive solutions such as the Kinect cannot produce sufficient depth resolution [[26], [27]] as required for characterising leaf veins. We further note that colour and topography of a surface are correlated in that topography changes will result in the incident light being reflected accordingly. This statement is intuitive and can be evidenced by various reflectance models, notably the Lambertian reflectance model. Therefore, we propose to explore topographic features as a way to improve detection accuracy and robustness. In the subsequent subsection, we introduce the principles of the Photometric Stereo method that can be employed to allow for analysis of leaf vein topography.

### 3.1 Photometric stereo principles

Photometric Stereo (PS) allows estimation of surface normals from reflectance maps obtained from images of the same object captured under different illumination directions. It was first introduced in [28] which illustrates that three views are sufficient to uniquely determine surface normal as well as albedo at each image point, provided that the directions of incident illumination are not collinear in azimuth. Other works employ four views for improved reconstruction performance [29]. PS techniques are superior in capturing detailed high-frequency 3D textures and are less affected by image noise compared to triangulation based techniques. In addition, PS methods normally require only one camera for image capture, simplifying the calibration process and allowing for high efficiency. In contrast, binocular stereo [30], for example, recovers depth of surface rather than surface orientations; which would likely introduce noise and artefacts.

Let$I_{1}\left(x,y\right),\;\;\;I_{2}\left(x,y\right)\;\;\;and\;\;\;I_{3}\left(x,y\right)$be three images captured under varied illumination directions. By varying the illumination direction, the reflectance map is changed accordingly, giving Eq. (1):

(1) $$\{\begin{array}{c} I_{1}\left(x,y\right)=R_{1}\left(p,q\right)\\ I_{2}\left(x,y\right)=R_{2}\left(p,q\right)\\ I_{3}\left(x,y\right)=R_{3}\left(p,q\right) \end{array}$$

A general reflectance map in gradient representation of the surface orientation and illumination direction is expressed in Eq. (2).

(2) $$R\left(p,q\right)=\frac{\varrho \left(1+pp_{s}+qq_{s}\right)}{\sqrt{1+p^{2}+q^{2}}\sqrt{1+{p_{s}}^{2}+{q_{s}}^{2}}}$$

where *ϱ* is the albedo,$\vec{N}=\left[-p,-q,1\right]$defines the surface normal vector, and$\vec{L}=\left[-p_{s},-q_{s},1\right]$defines the illumination direction. Let the surface bez=f(x,y), the gradients in the *x* and *y* directions become:

(3) $$\{\begin{array}{c} p=-\frac{\partial f\left(x,y\right)}{\partial x}\\ q=-\frac{\partial f\left(x,y\right)}{\partial y} \end{array}$$

These equations are derived under the assumptions that 1) the object size is small relative to the viewing distance; 2) the surface is Lambertian; and 3) the surface is exempt from cast-shadows or self-shadows. To simplify the expression, the light vector is further normalised to a unit vector$\vec{L_{n}}=\left[a_{x},a_{y},a_{z}\right]$. The relationship between a greyscale image and a reflectance map can also be written as:

(4) $$I\left(x,y\right)=\frac{\varrho \cdot \langle \vec{N},\vec{L_{n}}\rangle }{\vec{N}}=\varrho \cdot \frac{-pa_{x}-qa_{y}+a_{z}}{\sqrt{1+p^{2}+q^{2}}}$$

From Eq. (1) to (4), it is known that with three greyscale images$I_{1}\left(x,y\right)$, $I_{2}\left(x,y\right)$ and$I_{3}\left(x,y\right)$, along with three known light vectors$\vec{L_{n}{}_{1}}$, $\vec{L_{n}{}_{2}}$ and$\vec{L_{n}{}_{3}}$pointing in the directions of their respective light source, the surface normal and albedo at each image point can be uniquely determined.

### 3.2 Hardware system under a photometric stereo setting

**Fig. 1:**
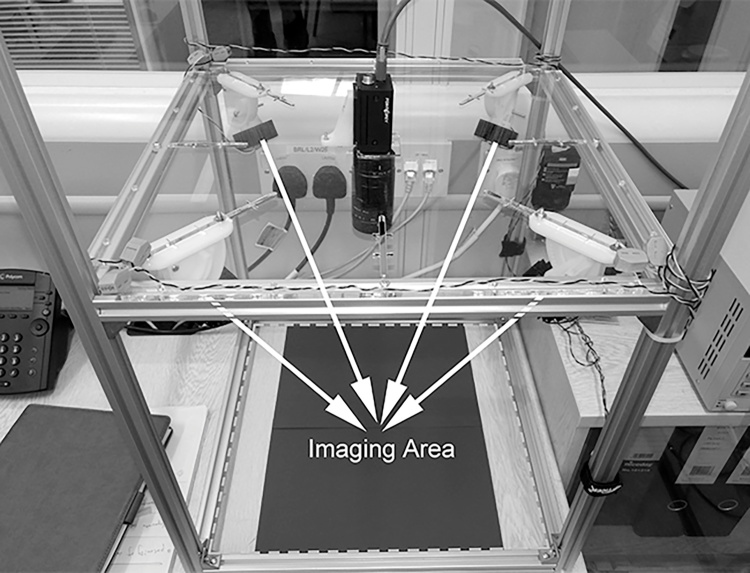
Overall structure of the PS 3D leaf imaging hardware system. The arrows represent active illumination from the four NIR illuminators.

The imaging system we designed consists of a colour camera, four Near-Infrared (NIR) illuminators with its matching band-pass filter and a synchronisation unit. The NIR illuminators have a wavelength of 940 nm. We demonstrate in the subsequent subsection the benefits of choosing this particular wavelength. We physically removed the IR cut-off filter located in front of the camera sensor such that NIR illumination beyond the visible spectrum can be captured. The synchronisation unit is controlled by an Arduino microcontroller which can switch on the illuminators alternately. A perfect synchronisation requires that a specific illuminator only remains lit for the whole duration of a camera exposure and that only one illuminator can be lit at a time. The structure of the system can be seen in Fig. 1.

In Fig. 1, it can be seen that the NIR illuminators are mounted symmetrically on the corners of the top panel and they point toward the centre of the imaging area at the bottom panel. The width and length of the system is 0.44 m and its height is adjustable to a maximum of 0.60 m. All the system components are inexpensive and available off the shelf, thus providing a low-cost 3D imaging solution. In addition to this, its structure can be conveniently scaled up and down, so that the system can be tailored to imaging from a few metres away or at a microscopic level.

Insufficient ambient illumination is often a limiting factor for the majority of imaging systems that are intended to work in real-world environments. In contrast, dark environments bring PS to its best performance by increasing the signal-to-noise ratio since ambient lighting accounts for noise in PS images. However, due to PS being active illumination based, strong ambient light will have a negative impact on generation of reflectance maps and thus on inference of surface normals. This is one of the main reasons that PS studies are commonly confined to well-controlled laboratory environments. We overcome this by applying an appropriate optical filter to selectively transmit light of a specific wavelength. Practically, the proposed PS system employs a 940 nm band-pass filter (with full width at half maximum being 10 nm) installed in front of the camera sensor such that the majority of ambient illumination can be blocked. The reason for choosing 940 nm illuminators is that common machine vision cameras can have desirable quantum efficiency at 940 nm (i.e. high power of signal) and that the 940 nm region of solar spectrum is much weaker in magnitude than its neighbouring range (i.e. low power of noise). As a result, high signal-to-noise ratio can be maintained.

### 3.3 3D leaf reconstruction

**Fig. 2:**
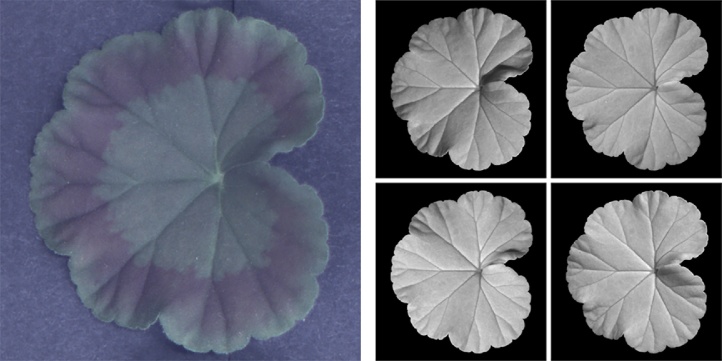
A geranium leaf captured as a single 2D image in (a) and as a PS image set in (b).

We introduced in preceding subsections the algorithm and device employed for 3D leaf imaging. In this subsection, we further demonstrate PS data acquisition and 3D reconstruction by using a geranium leaf as an example. Geranium leaves have inconspicuous veins as they do not cause much variation in colour or in topography to leaf surfaces when viewed from the upper side. They can also have a colour pattern that creates false edges, meaning that they pose great challenges to the leaf venation extraction task. This is why we choose a geranium leaf for this example. A geranium leaf image can be seen in Fig. 2(a) and the corresponding PS data captured by our PS imaging system (after subtracting ambient illumination and removing background) can be seen in Fig. 2(b).

**Fig. 3:**
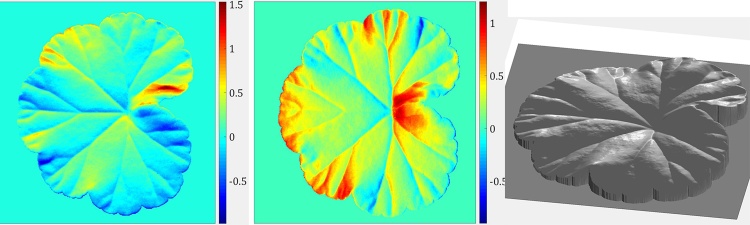
Recovered 3D features of the geranium leaf surface: surface gradient maps in (a) and the depth map in (b).

For acquisition of every PS image set, we capture images of a leaf illuminated under the four NIR illuminators as well as an ambient image (when all NIR illuminators are switched off), producing five images in total. The ambient image is then subtracted from the other four images. It should be noted that this paper is not dedicated to developing leaf segmentation algorithms and that our emphasis is on analysis of venation on segmented leaves. In this example and the rest of the paper, in order to obtain individual leaf foreground, we apply Otsu’s global thresholding [31]. It can be observed in Fig. 2(b) that, apart from enabling PS reconstruction, utilisation of NIR lights can remove the colour pattern of the leaf by penetrating the leaf surface moderately. Although PS also has the capability of separating colouring from topography by recovering surface normals, use of NIR lights serves as a preliminary step and also partly removes specularities. The pre-processed PS image set can then be employed to recover surface normals according to PS principles. We show in Fig. 3(a) surface gradients in two perpendicular directions, which are the *x* and *y* components of surface normals when the *z* component is normalised to unity. We further generate an integrated depth map, shown in Fig. 3(b), using the algorithm in [32]. However, since this algorithm enforces surface integrability which will introduce errors to the recovered 3D features, we only use depth maps to assist with visualisation but we adopt surface gradient features in the proposed leaf venation extraction algorithm.

In the subsequent section, we propose a leaf venation extraction algorithm that utilises local shape measures derived from 3D surface gradients. We continue to use the same geranium leaf in our examples for the sake of consistency, unless otherwise specified. More results of leafs of other species can be seen in Section 5.

## 4 Leaf venation extraction: ridges and ruts

**Fig. 4:**
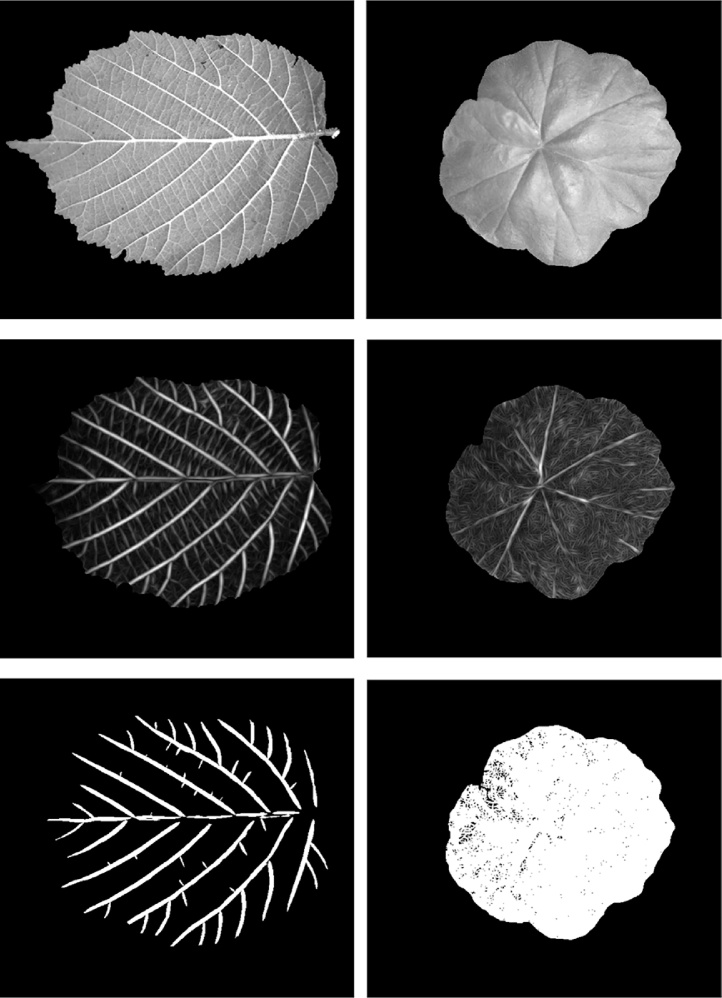
A comparison of leaf venation extraction on images of a hazel leaf and a geranium leaf, where true edges and false edges are present with varied magnitudes. The first row shows leaf regions with background removed. The second row shows Gabor response. The third row shows extracted leaf veins.

As discussed in the preceding sections, leaf veins can appear to be ridge-like or rut-like structures (i.e. positive veins or negative veins as we defined regarding leaf vein polarity). In fact, leaf veins are commonly modelled as edges in conventional explorations of colour or greyscale traits, as we reviewed in Section 2. This is not representative in that an edge is commonly marked by only one event of sharp changes in intensity values. In other words, a leaf vein with a given thickness should be characterised by a pair of edges where their gradient values should have opposite signs. Leaf venation extraction algorithms that rely on edges in colour or greyscale images are confounded by inherent diversity of leaves as well as dynamic environmental factors. In real-world environments, edges induced by different sources (i.e. true edges and false edges as we defined) are extremely difficult to be distinguished. Consequently, most existing leaf venation extraction methods have been only effective in dealing with cases where true edge responses are much stronger than false edges responses. However, true edge responses cannot always guarantee to produce salient features and therefore cannot be selectively preserved. To better explain this, we comparatively demonstrate how weak true edge responses can be overwhelmed by predominant false edges in greyscale images. In this example, we applied a leaf venation extraction method following [33] to a hazel leaf and a geranium leaf (different from the one used in the preceding section) captured under the same lighting condition – one with strong true edge responses from positive veins on the lower side of a leaf and the other with weak true edge response from negative veins on the upper side of a leaf. This comparison can be seen in Fig. 4.

**Fig. 5:**
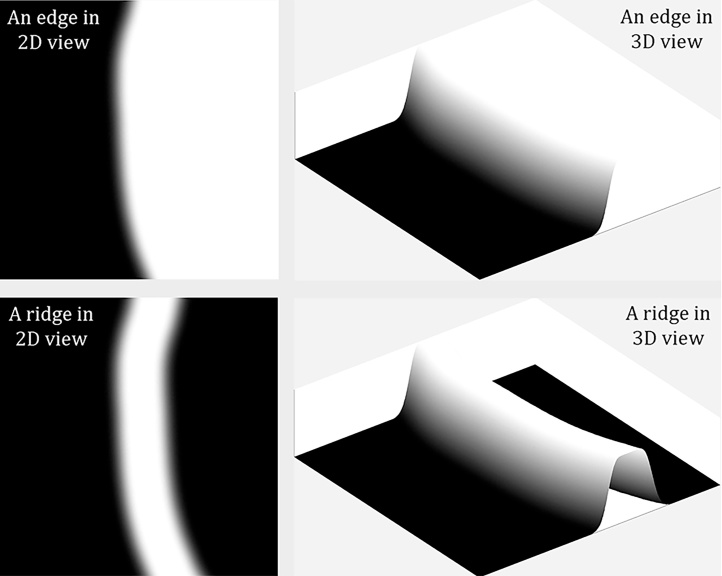
An illustration of modelling a leaf vein as a ridge as opposed to an edge, when visualised as a greyscale image or a 3D surface.

In Fig. 4, the first row shows two leaf images displaying strongly protruding vein structures or weakly recessed vein structures. The second row shows Gabor features extracted from the foreground leaf images. A filter bank consisting of Gabor filters with ten rotations is created for Gabor feature extraction. The last row shows leaf veins in a binary form obtained by applying Otsu’s thresholding method. It can be seen that when trues edges are not prominent enough to overcome false edges, it becomes extremely challenging to preserve leaf vein structures without excessive noise (i.e. false edges). Therefore, we propose to extract illumination-independent features that can minimise the effect of false edges and can emphasise 3D ridges and ruts. Fig. 5 illustrates why ridges and ruts are better than edges in modelling leaf veins.

As can be seen in Fig. 5, a ridge offers a more accurate and unique representation of a leaf vein and the same can be argued for a rut (which is an inversed ridge). Therefore we propose to utilise local shape and curvature measures for 3D ridge and rut detection.

### 4.1 3D ridge and rut detection by shape index and curvedness

**Fig. 6:**
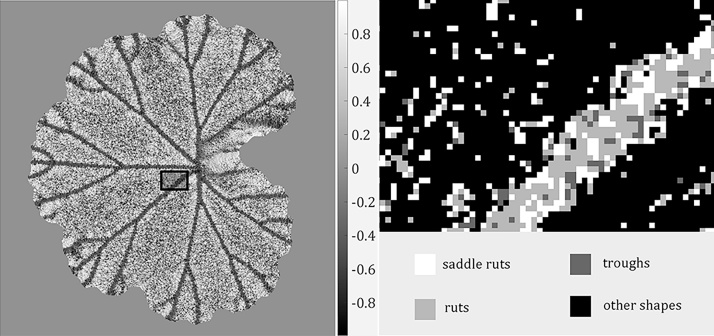
Shape index map and deviated shapes. (a) An example of shape index map. (b) An enlarged region of the shape index map that shows how ruts, troughs and saddle ruts coexist on a leaf vein segment.

Principal curvatures [34] represent the local maximum and minimum bending of a regular surface in a 3D Euclidean space. They have been known in the literature as decisive parameters that can fully describe local surface shapes. The Gaussian curvature, mean curvature, shape index and curvedness, derived by numerical operations of principal curvatures, have also been used as local shape measures. In this paper, we propose to utilise the shape index and curvedness for their capabilities to decouple local shapes and their sizes. This not only allows different local shapes to be distinguished from one another, but it employs topographical magnitude of a shape to remove additional noise. Due to limited space here, readers are referred to the work [35] for detailed definition and comparison of the aforementioned local shape measures, in particular, the shape index and curvedness. Nevertheless, we introduce in this section their concepts in brief but put a focus on how the two independent indicators can be united to produce a novel leaf vein indicator. The shape index is defined as:

(5) $$s=\frac{2}{\pi }\tan^{-1}\frac{k_{2}+k_{1}}{k_{2}-k_{1}}$$

where *k*_1_ is the maximum principal curvature of a surface and *k*_2_ the minimum. It can be seen from Eq. (5) that all non-planar surface patches can be mapped to s∈[−1,+1]. We explained in Section 3 that a dense surface normal map (i.e. surface gradients *p* and *q* in perpendicular directions) could be obtained by PS. Therefore, principal curvatures *k*_1_ and *k*_2_ can be derived from the gradient maps following [36], which further lead to the calculation of the shape index *s*. We visualise in Fig. 6(a) an example of shape index map. This result shows that most leaf veins obtain similar shape index values, meaning that leaf veins represented by the shape index can be characterised as discriminative local shapes from other leaf structures. According to [35], different shape index values can be mapped into nine shape categories including *spherical cup*, *trough*, *rut*, *saddle rut*, *saddle*, *saddle ridge*, *ridge*, *dome* and *spherical cap*. For example, a rut structure will have a shape index value s∈[−0.625,−0.375). Readers are referred to [35] for the mapping and visualisation of these shapes.

Although a group of leaf veins can be perceived as having the same 3D shape, in practical cases they commonly consist of a predominant shape as well as those in its neighbouring categories. For example, a rut structure can have pixels that appear to belong to the trough or the saddle rut category. This is demonstrated by an example in Fig. 6(b) which is an enlarged region (with a different colour mapping) within the black bounding box in Fig. 6(a).

To address this, rather than simply removing all non-rut pixels and resulting in the shape index map becoming very pixelated, we design a smooth function (Eq. (6)) to convert a shape index map into a weight map where the weight at a pixel location decreases when the corresponding local shape deviates from a rut structure.

(6) $$s_{w}=\{\begin{array}{cc} \sqrt[{}]{\frac{1}{1+{\left(\frac{\text{s}-{\text{s}}_{m}}{\omega }\right)}^{2\sigma }}}, & s<0\\ 0, & s\geq 0 \end{array}$$

**Fig. 7:**
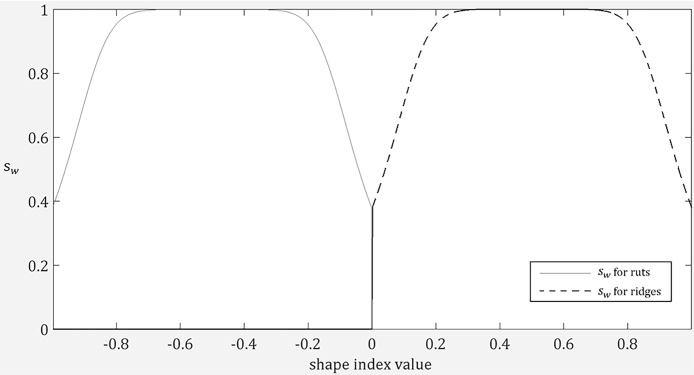
Response curves for positive and negative veins from shape index weight functions.

As can be seen in Eq. (6), this function emulates the frequency response of a Butterworth low pass filter. *s_w_* is the weight value calculated for each pixel location, *s* is the shape index value, *s_m_* is the median value of the shape index range for rut structures (i.e. s_m_ = − 0.5). σ and *ω* correspond to the order and the cut-off frequency of the filter and they control the flatness band and the roll-off rate of the resulting curve. As positive shape index values represent convex shapes, we set the corresponding weights to zeros. Note that the weight function for ridge structures is only slightly different in that *s_m_* = 0.5 and that concave shapes (instead of convex shapes) will have zero weights. Therefore, an intuitive rule is that, concerning any negative vein segment, ruts should have maximised weights, troughs and saddle ruts should have lower weights and all other concave variations should have rapidly decreasing weights. We found in our experiments that *σ* = 4 and *ω* = 0.4 produce a curve (shown in Fig. 7) that is in line with this rule and that yields the best performance.

With the *s_w_* map serving as a local shape measure, we continue to explore the curvedness as a size (i.e. topographic magnitude) measure. Similar to the shape index, the curvedness is also defined as a function of the principal curvatures:

(7) $$c=\sqrt{\frac{{\left(k_{1}+k_{1}\right)}^{2}}{2}}$$

**Fig. 8:**
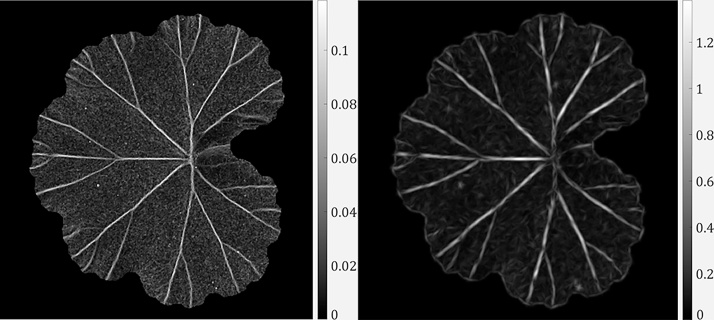
The curvedness response in (a) and its Gabor response in (b).

Therefore, the curvedness naturally describes the amount of surface curvature, which is always a positive value. Once a curvedness map is obtained, we apply an array of Gabor filters to it and produce a Gabor response map (following the same way as in the example in Fig. 4). In [33], use of Gabor filters showed to be effective in capturing leaf vein edges in colour images and in the absence of severe false edges. We use Gabor filters on the curvedness response map where most false edges have been removed. An example of the curvedness image and its Gabor response is shown in Fig. 8.

Then we take the dot product of the *s_w_* map with the Gabor response map ***G*** as the venation response map ***V***, formulated in Eq. (8).

(8) $$V=s_{w}\cdot G$$

**Fig. 9:**
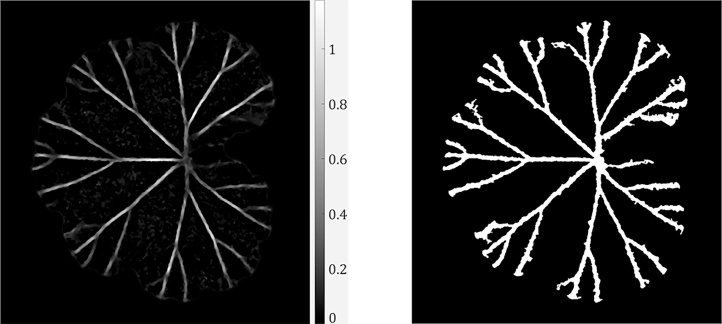
Venation response map in (a) and the extracted venation in (b).

Note that leaf polarity needs to be determined, so that the matching *s_w_* (for either ridges or ruts) can be used in Eq. (8). We will introduce in the subsequent subsection how leaf polarity can be determined. Once the venation response map is obtained, it is then converted to a binary image using Otsu’s method for thresholding. Small disconnected regions are removed to yield the extracted leaf venation. We show in Fig. 9 the venation response map and the extracted leaf venation.

The proposed algorithm can not only extract leaf venation, but it has the ability to reveal leaf vein polarity owing to utilisation of 3D features.

### 4.2 Determining leaf vein polarity

**Fig. 10:**
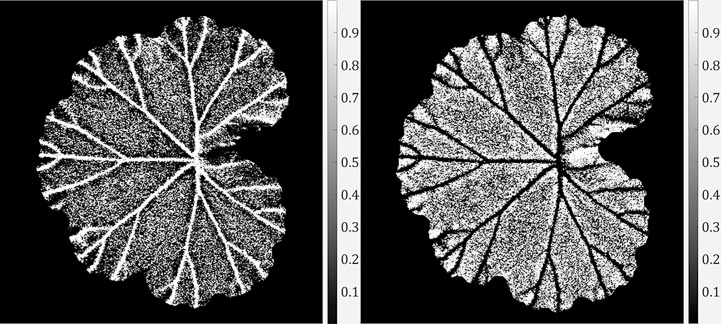
Shape index weight maps that match or mismatch the true vein polarity, shown in (a) and (b), respectively.

We remind the readers that we designed the *s_w_* functions for positive and negative veins (i.e. ridges and ruts) respectively, which are symmetrical to line *s* = 0. Without prior knowledge of vein polarity, there exist two solutions for the shape index weight map *s_w_*. The weight function that matches the true vein polarity will yield high values at vein locations where the mismatched weight function will give close to zero values. To demonstrate this, an example of the pair of *s_w_* maps is shown in Fig. 10.

**Fig. 11:**
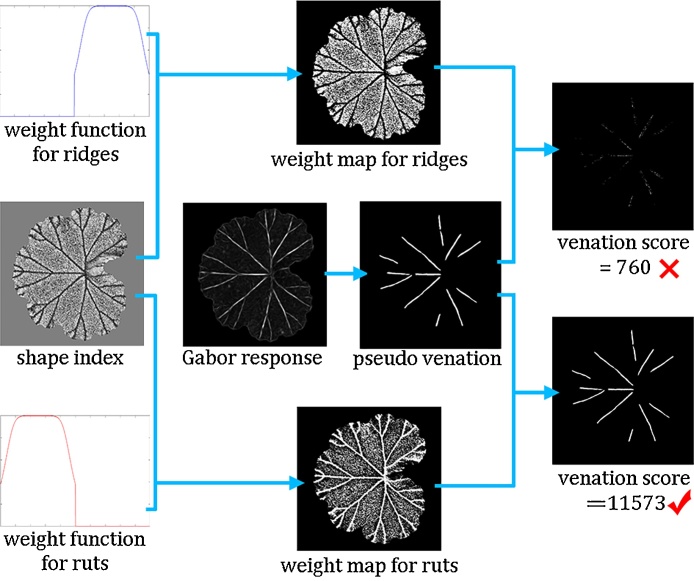
An illustration for determining leaf vein polarity.

Then we extract a *pseudo venation* directly from the Gabor response by Otsu’s method (i.e. an intermediate venation extraction result rather than that generated from the venation response map), where the threshold for segmentation is slightly increased to ensure that most non-vein pixels are removed. For each shape index weight map, we calculate its dot product with the pseudo venation and summate all pixel values into a venation score. The shape index weight map that yields the higher score is then the one that matches true vein polarity. This process is illustrated in Fig. 11.

In the next section, we report more experiment results on different leaf species in different conditions, e.g. healthy or diseased leaves, to demonstrate the robustness of the proposed method and its wide applicability.

## 5 Experiments and results

In this section, we show that the proposed method can be applied to leaves of different species where the venation architectures can be very distinctive. Our examples include geranium, tomato, mint and sage leaves, which are employed to illustrate the applicability of the proposed algorithm to palmate venation, pinnate venation, transverse venation and reticulated venation, respectively.

**Fig. 12:**
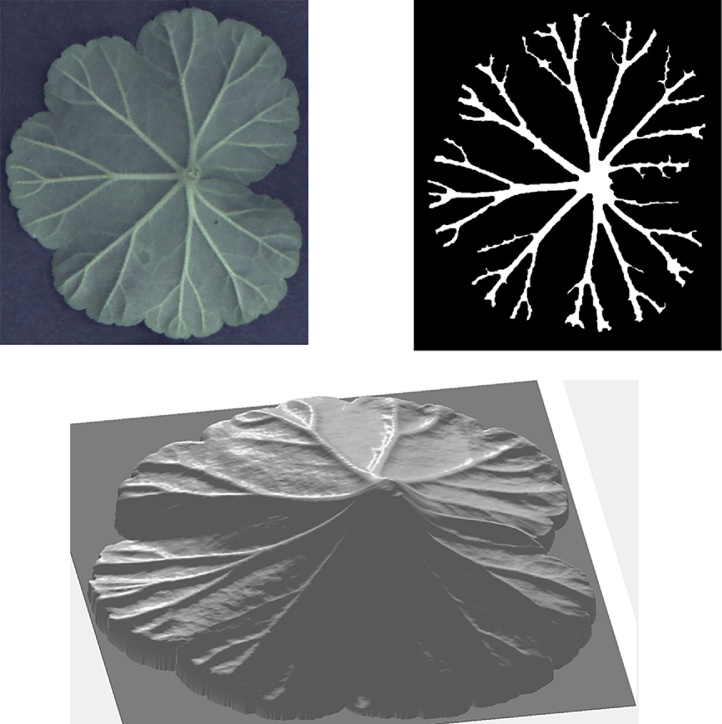
Leaf venation extraction on the lower side of the geranium leaf.

In each example, we visualise the colour/greyscale image, the extracted leaf venation and the reconstructed depth image from PS data. Note that all leaves employed were not manually flattened but they preserved natural local curvatures at the time of imaging. In Fig. 12, we show results of leaf venation extraction on the lower side of the geranium leaf demonstrated in preceding sections.

**Fig. 13:**
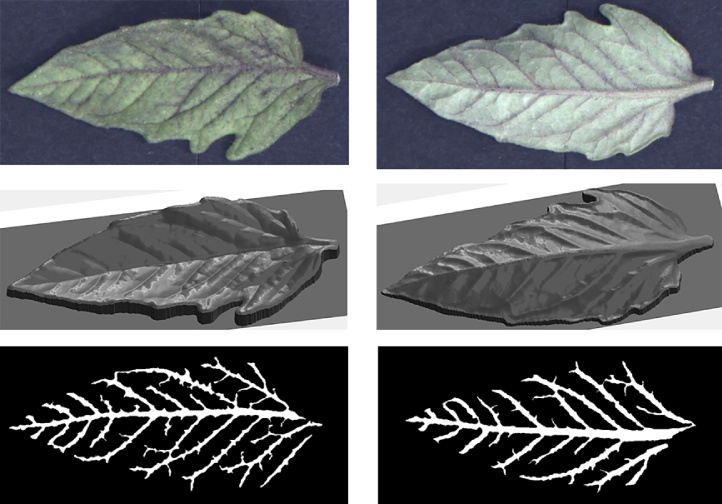
Leaf venation extraction on both sides of a tomato leaf.

**Fig. 14:**
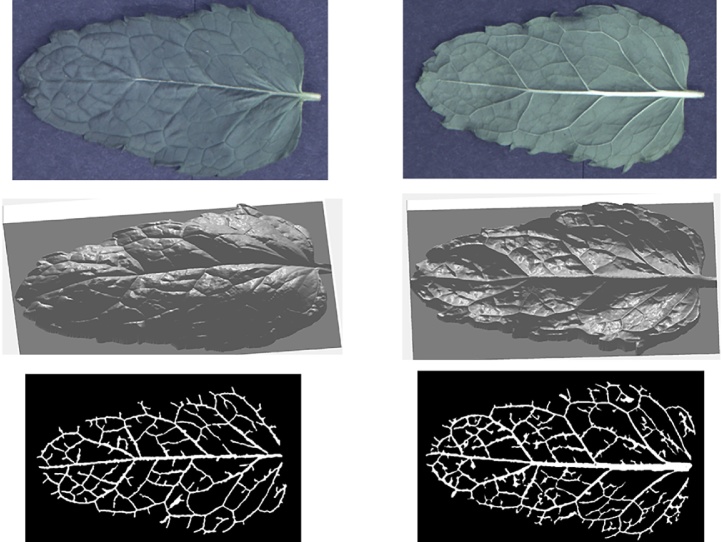
Leaf venation extraction on both sides of a mint leaf.

**Fig. 15:**
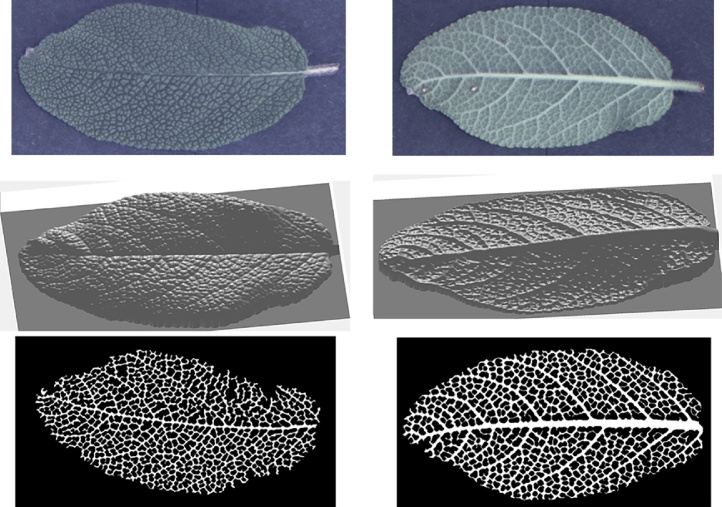
Leaf venation extraction on both sides of a healthy sage leaf.

**Fig. 16:**
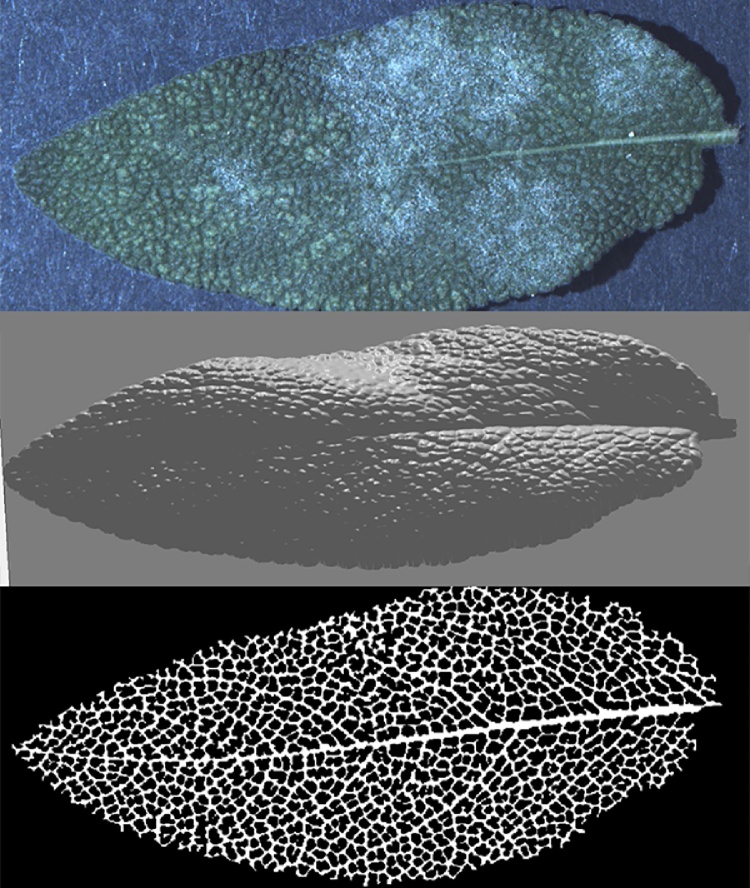
Leaf venation extraction on the upper side of a sage leaf with mildew infection.

More results can be seen in Fig. 13, Fig. 14, Fig. 15, Fig. 16, where leaf venation extraction was performed on a tomato leaf, a mint leaf, a sage leaf and a sage leaf with mildew infection.

The results again prove that the proposed algorithm is capable of extracting illumination-dependent features which can decouple surface colouring and surface topography. Due to this advantage, the proposed method extracted leaf venations accurately on the healthy leaves as well as the diseased sage leaf where dramatic changes in colour did not interfere with the salient shape index and curvedness features employed. In addition to leaf venation extraction, we calculated venation scores in each experiment, which were then utilised for determining venation polarity. These results can be seen in Table 1.

**Table 1:** Using venation scores to determining leaf vein polarity on both sides of leaves of different species. The higher score of the pair in each experiment is displayed in bold, which determines if venation patterns are ridges or ruts, i.e. positive or negative veins.

Leaf species	Leaf side	Venation score for ridges	Venation score for ruts	Vein polarity
Geranium	upper	760	**11573**	negative
Geranium	lower	**14278**	5044	positive
Tomato	upper	883	**6141**	negative
Tomato	lower	**13323**	2623	positive
Mint	upper	4321	**13481**	negative
Mint	lower	**14694**	6358	positive
Sage	upper	28419	**44242**	negative
Sage	lower	**26701**	17191	positive
Sage (diseased)	upper	40126	**76862**	negative

It can be seen from Table 1 that all vein polarities have been correctly recognised as positive venation or negative venation.

## 6 Conclusions

In this paper, we propose a PS based 3D imaging system together with a compatible and novel algorithm for accurate and robust leaf venation extraction. Our research aims at resolving challenges and bridging gaps in this area, which leads to a synergistic investigation of 3D image system and data acquisition, modelling of leaf venation, and robust and salient feature extraction. More specifically, active NIR lighting with a matching optical filter is employed to overcome undesirable illumination; the PS technique is adopted to inspire hardware customisation; leaf venation is modelled as 3D ridges and ruts as opposed to conventional colour-induced edges; surface normals are recovered as illumination-independent features where the shape index and the curvedness features are further derived and integrated such that local curvatures in dense 3D feature maps can be measured. By making 3D features available, the proposed method can also determine leaf polarity which reveals whether leaf venation is ridge-like or rut-like.

To demonstrate the applicability of the proposed method to different plant species, experiments were conducted on geranium, tomato, mint and sage leaves where leaf venation architectures are complex, subtle and varied. In contrast to the majority of conventional methods which can only be applied to the lower side of leaves where venation appears to be prominent and raised, the proposed method proves to be highly accurate by successfully dealing with both sides of leaves with varied venation architectures. We further showed that, by using a diseased sage leaf as an example, the proposed method reliably overcame dramatic colour changes, meaning that the seemingly subtle venation features extracted are in fact salient and robust. In all nine experiments, venation polarity recognition was achieved, which is beyond the capabilities of 2D feature based methods.

By making high-resolution and illumination-independent 3D features available, the proposed method is widely applicable to real-world environments and it can promise to drive forward many applications. For example, position of a meristem can be inferred from extracted leaf veins. This means that non-systemic herbicides (e.g. diquat) may be targeted in low dosage directly onto weeds, thereby yielding a more economical weed control solution while reducing environmental burdens. Another example concerns using leaf venations to assist with identification of plants that may be affected by viruses (e.g. mosaic virus in cassava). These potential uses of leaf venation will be explored in our future works.
